# A Review of Biogenic Volatile Organic Compounds from Plants: Research Progress and Future Prospects

**DOI:** 10.3390/toxics13050364

**Published:** 2025-04-30

**Authors:** Rongrong Luo, Xiaoxiu Lun, Rui Gao, Le Wang, Yuan Yang, Xingqian Su, Md Habibullah-Al-Mamun, Xiaohang Xu, Hong Li, Jinjuan Li

**Affiliations:** 1 College of Resources and Environmental Engineering, Guizhou University, Guiyang 550025, China; 18285485626@163.com (R.L.); wangle0602@foxmail.com (L.W.); 2 College of Environmental Science and Engineering, Beijing Forestry University, Beijing 100083, China; lunxiaoxiu@bjfu.edu.cn; 3 State Key Laboratory of Environmental Criteria and Risk Assessment, Chinese Research Academy of Environmental Sciences, Beijing 100012, China; gaorui@craes.org.cn; 4 Guizhou Research and Designing Institute of Environmental Sciences, Guizhou Academy of Environmental Science and Design, Guiyang 550081, China; gzhky_yy@163.com; 5 North Alabama International College of Engineering and Technology, Guizhou University, Guiyang 550025, China; 19078812302@163.com; 6 Department of Fisheries, Faculty of Biological Sciences, University of Dhaka, Dhaka 1000, Bangladesh; almamunhabib@gmail.com; 7 Key Laboratory of Karst Georesources and Environment, Ministry of Education, College of Resources and Environmental Engineering, Guizhou University, Guiyang 550025, China; xuxh@gzu.edu.cn

**Keywords:** biogenic volatile organic compounds (BVOCs), air pollution, ozone (O_3_), secondary organic aerosol (SOA), sampling analysis, reaction mechanisms

## Abstract

Biogenic volatile organic compounds (BVOCs) emitted by plants contribute to secondary air pollution through photochemical reactions in sunlight. Due to the influence of multiple factors, accurately characterizing and quantifying the emission of BVOCs from plant sources is challenging, which poses significant obstacles to the effective management and control of BVOCs. Therefore, this paper summarizes the emission mechanisms of BVOCs from plants, explores the primary factors influencing variations in the emission rates of these compounds, and evaluates the advantages and limitations of contemporary “measurement-modeling” methods for characterizing BVOC emissions. It is concluded that current measurement techniques still need to be further developed to meet the criteria of simplicity, affordability, and high precision simultaneously, and in terms of modeling and prediction studies, there is a lack of in-depth research on the atmospheric chemistry of BVOCs and the synergistic effects of multiple factors. Finally, it is suggested to leverage interdisciplinary strengths to develop advanced measurement technologies and high-resolution models for monitoring volatile compounds. Additionally, strategically selecting low-BVOC tree species in pollution-vulnerable urban areas—contingent on rigorous ecological assessments—combined with stringent controls on anthropogenic precursors (e.g., anthropogenic volatile organic compounds (AVOCs)) could serve as a complementary measure to mitigate secondary pollution.

## 1. Introduction

As public awareness of environmental protection increases, attention to environmental issues has intensified, particularly concerning air pollution, which directly affects human health. Among various air pollutants, volatile organic compounds (VOCs) stand out not only for their potential toxicity but also for their propensity to react with other pollutants, leading to secondary pollution. In 1990, the U.S. enacted the Clean Air Act Amendments, which listed 189 hazardous air pollutants (HAPs), including anthropogenic volatile organic compounds (AVOCs) such as benzene and toluene. The subsequent European Union Industrial Emissions Directive (2010/75/EU) also stipulates that industries emitting VOCs must implement Best Available Technology (BAT) to achieve a 60–90% reduction rate. And China also began to regulate VOCs at the beginning of the 21st century with the promulgation of the Volatile Organic Compounds (VOCs) Emission Control Standard. However, significant challenges remain for VOCs in non-regulated areas and emerging emission sources. Notably, biogenic volatile organic compounds (BVOCs) account for 90% of global VOC emissions [1,2,3]. The primary source of global BVOCs is plants, which naturally emit these compounds. However, their photochemical derivatives, like ozone (O_3_) and secondary organic aerosols (SOAs), may pose environmental and health risks in regions with elevated anthropogenic influences [4]. Although emissions from individual plants are relatively minor, forests, grasslands, wetlands, and agricultural lands—covering 31% of the Earth’s land surface—substantially amplify BVOC emissions [5,6,7]. Therefore, understanding the emission mechanisms of BVOCs and their synergistic effects with anthropogenic pollutants is critical for developing integrated pollution prevention and control strategies.

BVOCs are the main precursors of ozone (O_3_), accounting for 20% of total O_3_ production [8]. In addition, due to the photosynthetic characteristics of plants, the photochemical reaction activity of plant-generated BVOCs is significantly higher than that of anthropogenic volatile organic compounds (AVOCs) [9,10]. Under sunlight, BVOCs readily undergo photochemical reactions, contributing to the formation of biogenic secondary organic aerosols (BSOAs), which play a significant role in the global secondary organic aerosol (SOA) budget [11,12,13,14]. Although BVOCs are naturally emitted and not directly anthropogenic, their photochemical derivatives—tropospheric ozone (O_3_) and secondary organic aerosol (SOA)—are recognized as hazardous pollutants under elevated anthropogenic influences. Furthermore, a comprehensive understanding of BVOCs is vital for advancing insights into Earth’s ecological and atmospheric systems.

The historical evolution of BVOCs reveals significant progress driven by advancements in mechanistic studies and measurement techniques. In the 1950s, Sanadze [15] first observed the emission of gaseous organic substances from plants, initially identifying isoprene as a key compound [16]. With the application of gas chromatography, it became evident that BVOCs include a diverse array of around 30,000 hydrocarbons and oxides, including isoprene (C_5_H_8_), monoterpenes (C_10_H_16_), sesquiterpenes (C_15_H_24_), and other BVOCs [17,18,19]. These compounds exhibit diverse chemical structures and properties, necessitating specialized detection methods for their identification and quantification. By gas chromatography–mass spectrometry (GC-MS) technology, Kesselmeier and Staudt [20] provided insights into BVOCs biosynthesis, emission inventories, and the relationship between emissions, plant physiology, temperature, radiation, and ecophysiological functions. Helmig et al. [21] further advanced BVOCs detection by employing thermal desorption–gas chromatography–mass spectrometry (TDS-GC-MS), which enabled the analysis of a broader range of polar compounds in BVOCs and facilitated deeper mechanistic studies of BVOCs.

Despite these advances, challenges still persist in detecting and identifying the types of BVOCs from plants. Atmospheric concentrations of BVOCs from plants are typically very low, and their high reactivity leads to rapid chemical transformation or adsorption within sampling equipment, complicating accurate measurement. Additionally, temporal and spatial variations in BVOC emissions—driven by factors such as industrial activity, regional environmental differences, and seasonal climate changes—introduce further complexities [22]. On larger scales, studies by Papiez et al. [23] and Kramshøj et al. [24] revealed that cumulative emissions of isoprene, monoterpenes, and other BVOCs, amplified by anthropogenic climate perturbations, contribute to atmospheric processes (e.g., O_3_ formation) and carbon–climate feedback, thereby indirectly influencing climate warming trends.

Recent advancements in computational methodologies have revolutionized the study of BVOC emissions from vegetation. Research paradigms have transitioned from foundational investigations into biochemical mechanisms and empirical sampling analyses to the incorporation of advanced machine learning architectures. These computational innovations facilitate the predictive modeling of BVOCs’ multifactorial impacts, including climate adaptation dynamics, O_3_ generation pathways, and SOA formation kinetics. Guenther et al. [3,25,26] pioneered BVOC emissions modeling, developing global models that laid the foundation for quantifying BVOC emissions. On this basis, Pierce [27] studied the aerosol impacts on the climate systems by the improved atmospheric chemistry models. More recently, Ng et al. [28] explored interactions between nitrate radicals (•NO_3_) and BVOCs during SOA formation and highlighted the importance of such interactions in improving atmospheric model predictions. Tang et al. [29] combined dynamic vegetation models, such as LPJ-GUESS v4.1, with global chemical transport models like TM5-MP, demonstrating that the spatial pattern of the BVOCs-driven SOA impact was strongly influenced by climate-induced vegetation changes.

Now, the research on BVOCs is no longer limited to a single discipline. Studies by Lun et al. [30] and Yang et al. [31] have contributed significantly to initiating BVOCs research by reviewing sampling and analysis methods, emission inventories, and emission factors. As disciplines such as chemistry, biology, mathematics, and computer science intersect with environmental science, new perspectives in BVOCs from plant research are emerging. This paper reviews recent studies on BVOCs emission sources, chemical composition, and reaction mechanisms; discusses their environmental impact factors; and evaluates effective mitigation strategies. Additionally, it highlights future interdisciplinary research trends, providing valuable insights for addressing BVOCs-related atmospheric pollution.

## 2. Methodology

**Figure 1:**
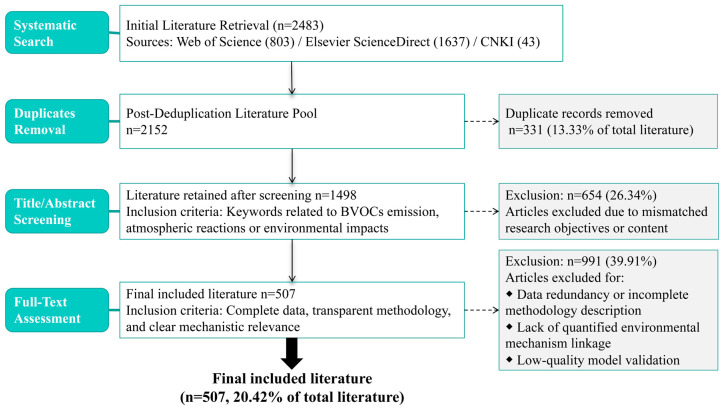
Narrative review flow diagram.

To comprehensively review and summarize the research progress of BVOCs, this study adopted an interdisciplinary narrative review framework (Figure 1). A systematic literature search was conducted across multiple databases, including Web of Science, Elsevier ScienceDirect, and the China National Knowledge Infrastructure (CNKI). Keywords such as “BVOCs”, “BVOCs emission”, and “atmospheric oxidation” were employed for thematic searches, yielding an initial collection of 2483 articles. Following the screening and analysis of search results, duplicate records were removed, resulting in 2152 articles selected for detailed evaluation. Subsequently, 654 articles unrelated to the research scope were excluded based on titles and abstracts. Finally, through full-text precision assessments, 991 articles were further excluded due to data redundancy, incomplete methodological descriptions, or lack of environmental mechanism relevance, ultimately retaining 507 research articles for in-depth analysis.

**Figure 2:**
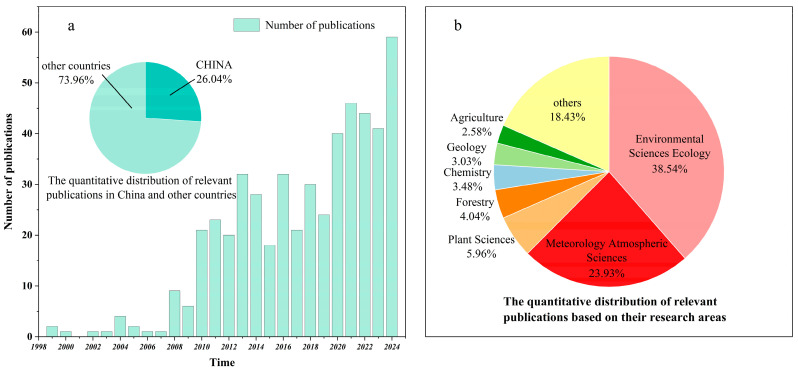
Distribution of 507 studies on biogenic volatile organic compounds (BVOCs) since 1999. (**a**) The change in the number of publications related to BVOCs in the world and the proportion of the number of articles published in China and other countries. (**b**) The quantitative distribution of relevant publications based on research areas.

As shown in Figure 2, the reviewed literature comprises 132 domestic Chinese articles and 375 international articles, spanning from 1999 to 2024. The earliest relevant articles originated in the United States, followed by contributions from South Korea. Between 1999 and 2009, only 28 articles were published. Since 2010, however, the number of publications has steadily increased, reaching an explosion in 2020 and reaching its peak in 2024. This trend highlights the growing global interest in BVOCs research, particularly in understanding their atmospheric interactions and environmental impacts.

## 3. BVOC Emissions Characterization Methods

### 3.1. Methods of Emission Measurement

#### 3.1.1. Single-Plant Scale Sampling Methods

To comprehensively understand the impact of BVOC emissions on atmospheric chemistry and the environment, it is essential to accurately monitor their emission flux. Currently, emission measurements of BVOCs from a single plant focus on the leaf scale, and the main methods are divided into on-line and off-line techniques (Table 1), with each category encompassing various measurement techniques and technologies, which involve analytical techniques as shown in Table 2.

(1) On-line measurement

On-line measurement methods are frequently employed for monitoring environmental conditions in the field. On-line detection can analyze the emission concentration of BVOCs in the atmosphere and their temporal variations in real time. This method typically involves direct air sampling and immediate analysis using advanced instruments [55,56,57].

Representative on-line measurement methods include proton transfer reaction–mass spectrometry (PTR-MS) [58,59], Fourier transform infrared spectroscopy (FTIR) [60,61], portable gas chromatography–flame ionization detector (GC-FID), and portable GC-MS [62,63]. PTR-MS is particularly useful for trace gas analysis and is widely used in outdoor atmospheric measurements. To study the response of temperate deciduous tree species to flooding stress, Copolovici et al. [64] used proton transfer reaction–mass spectrometer (PTR-MS, high sensitivity version) to measure VOC concentration. Gómez et al. [34] routinely determined VOCs and qualitatively identified monoterpene compounds in ambient air using a gas chromatography system with GC-FID. You et al. [65] upgraded the traditional portable gas chromatograph and used it for continuous and on-site determination of trace volatile organic compounds, especially aromatic volatile organic compounds, in air samples. 

The FTIR device can identify and quantify multiple BVOCs in air by measuring the infrared absorption spectrum of the gas without sample pretreatment and can simultaneously detect multiple compounds. Kalalian et al. [35] used FTIR and solid-phase microextraction sampling combined with a gas chromatography–mass spectrometry (SPME-GC/MS) in situ analysis of the reaction products of O_3_ and three different BVOCs to predict the formation of photooxidants such as SOA. In order to check the health of plants, Sharma et al. [66] developed and applied a fully automated portable gas phase color spectrum for a rapid and in situ analysis of BVOCs from plants. Cicciol et al. [67] measured the vertical fluxes of volatile organic compounds from vegetation in a Mediterranean forested area using PTR-MS.

In addition to these methods, electronic noses (E-noses) represent an emerging technology for real-time environmental monitoring. E-noses offer high sensitivity and rapid response and eliminate the need for the complex pretreatment processes associated with gas chromatography sensors [36,68]. Feng et al. [69] used E-noses, headspace solid-phase microextraction–gas chromatography–mass spectrometry, and headspace–gas chromatography–ion mobility spectrometry to identify and characterize volatile organic compounds in eight kinds of *Zanthoxylum bungeanum* with geographical indications in China, providing a useful basis for the study of the differences in floral aroma. However, while E-noses are advantageous for qualitative and semi-quantitative analysis, the high complexity of environmental backgrounds can complicate the practical application of the E-noses, and their analytical accuracy and repeatability remain limited.

(2) Off-line measurement

Off-line measurement methods involve air sample collection and laboratory analysis. These methods are advantageous for their simplicity, high accuracy, and high sensitivity, but they lack real-time data.

(1) Sample collection

Off-line measurement methods separate sampling from analysis, where samples are collected on-site and then transported to a laboratory for analysis. Sample preservation methods for the different biogenic volatile organic compound (BVOC) collection containers are shown in Table 3. These methods are simple to operate, highly accurate, and sensitive. However, because samples need to be transported, they cannot reflect real-time data.

Typical collection containers for BVOCs include sampling bags (such as Tedlar^®^ Bags and Teflon Bags), adsorption tubes (such as Tenax^®^ Tubes and Activated Charcoal Tubes), and sampling canisters (such as SUMMA Canisters, Stainless Steel Canisters, and Silonite Canisters). Of these, the most commonly used containers for ambient air sampling are SUMMA Canisters, which have special treatments such as silanization or fused silica coatings that minimize the adsorption or reaction of VOCs on the walls of the canisters, thus maintaining the chemical stability of the sample. Sampling methods mainly include static closed sampling and dynamic headspace sampling. Static closed sampling, a classic method for calculating gas emission rates, involves measuring the concentration of BVOCs in a chamber or bag containing the plant species over time to determine the emission rate. Lin et al. [70] demonstrated that static enclosure techniques and in situ sampling can accurately capture BVOC emissions from entire leaves, which can then be estimated using remote sensing data. To address the limitations of static enclosure techniques, a semi-static method has been developed. Li et al. [71] conducted repeated experiments to evaluate the performance of the semi-static method for measuring BVOC emissions, confirming the feasibility of semi-static closed techniques as a screening tool for characterizing monoterpene emissions.

While static methods allow for rapid measurements and lower detection limits, they are susceptible to environmental conditions; factors such as temperature, pressure, and humidity can easily alter the equilibrium of BVOCs between the sample and the gas phase [72]. To address these limitations and meet the measurement requirements of various different scenarios, researchers have developed a dynamic headspace sampling method. This dynamic system features air circulation capabilities, effectively reducing discrepancies in environmental parameters inside and outside the system, enabling comprehensive environmental control for accurate BVOCs emission measurements. Vedel-Petersen et al. [73] utilized dynamic enclosure techniques to collect BVOC emissions from Arctic plants; this approach reduced environmental influence and allowed for the study of emission characteristics and seasonal variations in BVOCs. Jing et al. [74] also used dynamic headspace sampling to investigate BVOC emissions in northern China, concluding that different tree species emit different components at different rates, further demonstrating the advantages of dynamic headspace sampling. Dynamic headspace sampling is the most commonly used method for measuring BVOC emissions from living plants.

(2) Sample analysis

After field samples are collected, they are preserved and transported to the laboratory using specific methods to maintain sample integrity, as shown in Table 3. Once in the laboratory, these samples must typically be analyzed using various detection instruments within a specific timeframe to ensure accurate results.

At present, the most widely used separation and identification instrument is GC-MS; TDS-GC-MS is often used in the qualitative analysis study of BVOC emissions. Yuan et al. [75] quantified isoprene emissions from young hybrid poplar trees using TDS-GC-MS, and for the first time, investigated the combined effects of O_3_ and drought on isoprene emissions. In order to detect the main BVOC species emitted by Mediterranean plant species, Fattobene et al. [38] employed thermal desorption gas chromatography–mass spectroscopy (TD-GC-MS) to analyze the air on-site sampled in the field.

Based on GC-MS analysis methods, researchers have also improved and developed automatic thermal desorption–gas chromatography–mass spectrometry (TCT-GC-MS), thermal desorption gas chromatography–time-of-flight mass spectrometry (TD-GC/TOF-MS) [40], etc.

**Table 3:** Sample preservation methods for different biogenic volatile organic compound (BVOC) collection containers.

Collection Containers	Pretreatment Requirements	Storage Conditions	Maximum Preservation Time	Applicable Compound Range	Ref.
Tedlar Bags	Nitrogen flushed 3 times, light-proof aluminum foil wrapped	4 °C, <30% humidity	48 h	Nonpolar terpenes (C_5_-C_10_)	[76,77]
Tenax Tubes	Aging (320 °C, 2 h), vacuum sealed in aluminum foil pouch	−20 °C with desiccant	7–30 days	Semi-volatile VOCs (C_10_-C_15_)	[78,79]
Activated Charcoal Tubes	Aged 1 h at 400 °C, sealed in moisture-proof container	4 °C, avoid condensation	14 days	Highly volatile VOCs (C_2_-C_8_)	[80,81]
SUMMA Canisters	Vacuum baked at 160 °C for 12 h, nitrogen filled to 30 psi	Protected from light at room temperature, pressure monitoring	30 days	Broad spectrum VOCs (C_2_-C_15_)	[77,82]

Typical collection containers for BVOCs include sampling bags (such as Tedlar^®^ Bags and Teflon Bags), adsorption tubes (such as Tenax^®^ Tubes and Activated Charcoal Tubes), and sampling canisters (such as SUMMA Canisters, Stainless Steel Canisters, and Silonite Canisters).

#### 3.1.2. Canopy Scale Flux Measurement Methods

The emissions of BVOCs exhibit high spatiotemporal heterogeneity. Traditional leaf-scale studies are constrained by the limited spatial representativeness of sampling methods. However, canopy-scale flux measurement methods such as Eddy Covariance (EC) and Relaxed Eddy Accumulation (REA), which directly capture integrated canopy-level fluxes, significantly enhance spatial representativeness, temporal continuity, and data comprehensiveness [83,84]. While early EC technology provides direct turbulent flux data, its reliability for BVOCs—due to their ultra-low concentrations and adsorption losses—requires advanced sensors like PTR-MS [85]. For example, Spirig et al. [86] combined EC and PTR-MS to measure BVOCs canopy fluxes in a mixed deciduous forest in northwestern Germany, validating EC’s reliability under complex heterogeneous canopy conditions, with isoprene and monoterpenes identified as dominant emissions. Misztal et al. [87] employed EC-PTR-MS to directly quantify BVOC fluxes in Southeast Asian oil palm ecosystems, revealing isoprene’s predominant role (84% of midday fluxes) and significant contributions from floral emissions. To enhance signal-to-noise ratios, REA integrates airflow separation and offline analysis (e.g., GC-MS) for trace BVOC detection. Bai et al. [88] applied REA and gradient techniques to conduct long-term continuous canopy flux measurements of BVOCs in a subtropical pine plantation, uncovering pronounced diurnal, seasonal, and interannual variations in isoprene and monoterpene emissions.

Subsequent advancements include Disjunct Eddy Covariance (DEC), which combines EC’s turbulence-phase data with REA’s intermittent sampling, broadening EC’s applicability [89,90,91]. Emerging methods like Disjunct Eddy Accumulation (DEA) leverage ultra-low-frequency sampling and automated accumulation to reduce power consumption and costs, enabling long-term field observations [92]. Current trends focus on integrating multiple methods and utilizing Unmanned Aerial Vehicle (UAV) platforms to expand 3D flux monitoring capabilities, thereby comprehensively elucidating the spatiotemporal patterns of BVOC emissions and their impacts on atmospheric chemistry [93,94,95].

### 3.2. Model-Based Estimation of BVOCs Emission

Although canopy-scale flux measurement techniques provide high-precision real-time flux data through direct observation, effectively reflecting plant responses to environmental changes, this method has limitations in spatial coverage and long-term monitoring, making it unable to comprehensively capture the dynamic variations in BVOCs.

Emission models bridge critical knowledge gaps by synthesizing empirical observations with environmental drivers (e.g., meteorology and land use patterns), enabling dynamic predictions of BVOC fluxes across heterogeneous ecosystems and under varying climatic scenarios. These tools are particularly vital for scaling plot-level measurements to regional or global assessments, where direct monitoring becomes logistically impractical. The development of BVOC emission models serves a dual purpose: (1) to extrapolate mechanistic insights from laboratory and field studies into predictive algorithms, and (2) to inform policy-relevant scenarios such as air quality management and climate mitigation strategies.

**Figure 3:**
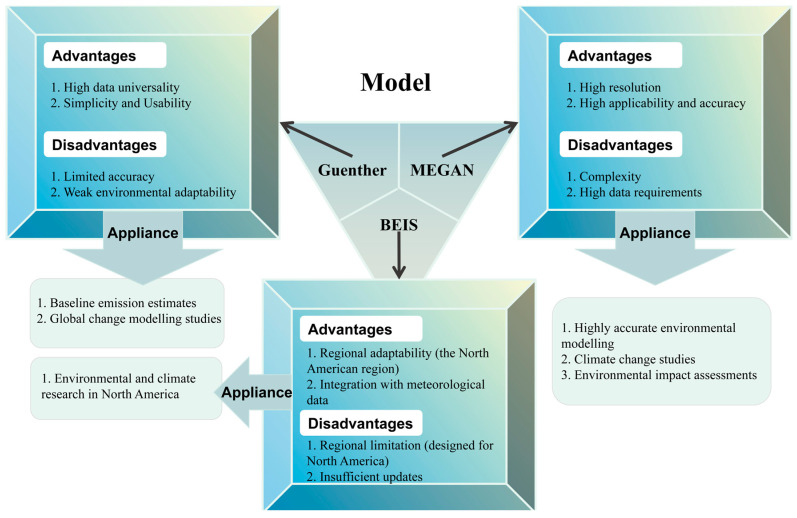
The characteristics of different model simulation methods of biogenic volatile organic compounds (BVOCs) and their applicable scenarios.

Thus, in the study of BVOC emissions in a certain area and at a large scale, the model-based estimation method is mainly used [96,97]. The model simulation method is based on remote sensing data to obtain the regional leaf area index and leaf biomass, combined with emission factors, meteorological data, and other information to use model simulation to obtain the total emission of plant-derived volatile organic pollutants, including the Guenther model (Guenther), model of emissions of gases and aerosol from nature (MEGAN), biological source emission inventory system model (BEIS), and so on. Their main characteristics and applicable scenarios are summarized in Figure 3. In recent years, the various models have been continuously upgraded and developed and are widely used in regional and global BVOC emission research.

#### 3.2.1. Guenther

The isoprene emission model-G91, constructed by Guenther et al. [26] based on the photosynthetic electron transport model developed by Farquhar and the physiological characteristics of plants, provides an important framework for estimating inventories of biogenic non-methane hydrocarbon emission rates. In order to simulate the effects of light and leaf temperature on the emission of plant isoprene and monoterpenes, Guenther et al. [25] developed G93 on the basis of G91. Subsequently, based on the G91 and G93 algorithms, Guenther et al. [3] developed a global natural VOCs emission model, G95, based on relevant data, which has a high-resolution spatial grid to estimate hourly averaged emissions of organic compounds from natural sources. Chi et al. [98] used the Guenther model to convert vegetation volume and yield data, such as the forest and grassland resource inventory and vegetation maps, into leaf biomass data to estimate the space–time and source distribution of the emissions of BVOCs in China, and established a BVOC emissions inventory in mainland China. In order to explore the emission of BVOCs in Qinghai Province and its impact on air quality, Wang et al. [99] used the G95 method to estimate the total emissions of isoprene, monoterpenes, and other VOCs emitted by vegetation in Qinghai Province in 2021.

#### 3.2.2. BEIS

Pierce et al. [27] established a biological emission inventory system model-BEIS1 based on the data of leaf biomass, land use, emission factors, and meteorological parameters obtained by observation or calculation to estimate VOCs emissions in vegetation and NO emissions in soil. Subsequently, Pierce et al. [100] developed the second version of the bio-emission inventory system BEIS2 on the basis of BEIS1. BEIS2 updated the land use inventory, emission factors, and environmental correction formulas following the G95 algorithm. Considering the factors such as plant growth environment, the National Center for Atmospheric Research (NCAR) and the United States Environmental Protection Agency (EPA) developed the global biosphere emission and interaction system—GloBEIS [30]. With the development of geographic information system technology, researchers have successively updated and developed BEIS3, BEIS4, etc. Feldman and Zheng et al. [101,102] used vegetation data and the GloBEIS model to estimate the total emission of plant-derived volatile organic compounds in southeastern Texas of the United States and in the Pearl River Delta of China.

#### 3.2.3. MEGAN

Based on the G95 model and multi-source information such as satellite and ground observation data, Guenther et al. [2] constructed the natural gas and aerosol emission model, MEGAN, to estimate global terrestrial isoprene emissions. This model is suitable for regional- and global-scale chemical and transport modeling. With the deepening of research, researchers have successively developed MEGANv2.02 [103], MEGANv2.1 [104], MEGANv3.0 [105], and so on.

Sindelarova et al. [106] used the MEGANv2.1 model and MERRA meteorological field to establish a monthly global BVOC emissions dataset from 1980 to 2010. The model estimated the average annual BVOC emissions to be 760 Tg (C) yr^−1^. Using the improved MEGAN-BEIS361 model combination based on the MEGAN model, Wang et al. [107] simulated and predicted isoprene emissions and environmental concentrations in the United States. Jiang et al. [108] simulated biogenic isoprene emissions using the MEGAN3 response to drought and its impact on global atmospheric chemistry. Compared to the Guenther model and BEIS models, the MEGAN model is more widely used and is one of the commonly used models.

The operation of these three models requires various types of remote sensing data, meteorological data, and emission factors based on laboratory or field observations. They are widely used to obtain BVOC emissions across different temporal and spatial scales, providing effective methods for large-scale BVOC research. In recent years, building upon these three major models, researchers have successively developed new models such as the empirical model of BVOC emissions (EMBE) [109] and the Plant Specific Emission Model (PSEM) [67], offering new approaches for BVOC emission studies.

## 4. Emission Mechanisms of BVOCs from Plants

### 4.1. Emission Sources

The sources of BVOCs are highly diverse, both in urban and rural environments. In urban areas, both natural vegetation remnants (e.g., preserved woodlands) and cultivated green infrastructure (e.g., parks and street trees) serve as dominant sources of BVOCs. Notably, in certain cities, the spatial coverage of landscaped vegetation surpasses that of natural ecosystems, which may generate a distinct urban BVOCs signature compared to natural landscapes. Furthermore, the synergistic effects of environmental stressors—particularly the urban heat island effect and industrial pollutants—alter the compositional characteristics (e.g., shifts in isoprene-to-monoterpene ratios) and elevate the emission fluxes of plant-derived BVOCs, thereby intensifying their role in urban atmospheric chemistry [110,111]. Jing et al. [74] investigated BVOC emissions from dominant forest vegetation species in Beijing, finding that urban forest trees primarily emit isoprene and monoterpenes. Bao et al. [112] estimated BVOC emissions from 23 urban plants in Shenzhen and discovered that BVOC emissions from urban green spaces account for approximately 20.07% of the total BVOCs in the study region, with their estimated emissions in built-up areas (1.65 kt) nearly doubling conventional estimates (0.86 kt). This result challenges the historical marginalization of urban vegetation in BVOC emission inventories.

In rural areas, natural forest vegetation, agricultural crops, and wetlands are primary BVOC sources. Forests, in particular, are the largest contributors, as their emissions are highly influenced by natural environmental conditions [104,113,114]. Smiatek and Steinbrecher [115] studied the temporal and spatial variation in VOC emissions from German forests and found that the average annual BVOC emissions from forests during 1994–2003 were 366 Gg. Liu et al. [116] identified isoprene and monoterpenes as the primary emissions from pine and birch forests, with these compounds presenting the highest BVOC mixing ratios in forest ecosystems. Bamboo forests are also a major BVOC source [117]. Okumura et al. [118] found that 86% of bamboo species emit isoprene at rates ranging from 0.7 to 99.1 nmol m^−2^ s^−1^, with *Phyllostachys glauca*, *Semiarundinaria fastuosa,* and *Bambusa pervariabilis* being prominent sources.

Wetland plants, such as *Phragmites australis*, *Typha angustifolia*, *Iris pseudacorus*, and *Cyperus rotundus*, also produce BVOCs [119,120]. In agricultural landscapes, crops such as wheat, corn, and canola can release BVOCs, with corn and canola emitting methanol and acetaldehyde [121,122]. Additionally, microbial activity in soil produces small amounts of BVOCs, which, while minor in magnitude, are significant on a global scale [123,124,125,126]. Wester-Larsen et al. [127] reported variation in soil BVOC (SBVOC) concentrations based on different vegetation types, with areas of high vegetation cover and biodiversity, such as the Amazon rainforest and tropical forests in Southeast Asia, exhibiting higher BVOC emissions [128,129,130].

In both urban and rural areas, human activities have solidified the plant species in those regions (landscape plants in urban areas and economic crops in rural areas). If the emission sources of BVOCs in human-affected areas are not taken into account and higher precision emission estimates are not employed, there will be significant deviations in the understanding of BVOC emission characteristics. Therefore, studies on BVOC emissions require regional representation, with global research necessitating multi-regional composite studies that account for vegetation cover and topography. This paper primarily focuses on plant-derived BVOC emissions.

### 4.2. Compositions

BVOCs are broadly categorized into four main groups: isoprene, monoterpenes, sesquiterpenes, and oxygenated biogenic volatile organic compounds (OBVOCs), which include alcohols, aldehydes, ketones, and esters (Table 4). Among these, isoprenes and monoterpenes dominate, accounting for over 70% of the total annual BVOC emissions, particularly during the summer in temperate forests [131]. Liu et al. [132] revealed that isoprene emissions from *Ficus virens* could account for up to 85% of the total plant emissions. Similarly, monoterpene from *Camphora officinarum* contributes over 75% to total emissions and is highly reactive in forming O_3_ and SOA. To further illustrate the variability in BVOC emissions and concentrations, we present Table 5, which provides a comparative analysis of the mean concentrations of key BVOC components, such as isoprene and monoterpenes, across different study areas and climatic conditions [133].

Although sesquiterpenes and oxygenated biogenic volatile organic compounds (OBVOCs) are emitted in smaller quantities, they still significantly influence atmospheric chemistry. Liu et al. [134] confirmed that 2-methyl-3-buten-2-ol and methanol from pine forests undergo rapid oxidation, leading to SOA formation. While sesquiterpenes are emitted in lower amounts compared to isoprene and monoterpenes, they dominate in specific plant species such as *Empetrum hermaphroditum* and *Cassiope tetragona* [135]. Sesquiterpenes exhibit high oxidation reactivity with hydroxyl radicals (OH) and ozone (O_3_), resulting in rapid degradation and short atmospheric residence times (minutes to hours). Nevertheless, their oxidation products are important precursors of secondary organic aerosols (SOAs) [136,137,138]. Similarly, OBVOCs, such as esters and ketones, have short atmospheric lifetimes. For instance, certain ketones (e.g., methyl vinyl ketone) exhibit lifetimes of less than 1 h, where their low persistence is governed more by photolysis than by “lower chemical reactivity”. This limits their direct contribution to ozone formation but may enhance nighttime nitrate radical (NO_3_) chemistry [139,140,141,142]. The rapid dissociation of various BVOCs, including isoprene [143], α-pinene [144], β-pinene [145], d-limonene [146], and linalool [147], plays a critical role in O_3_ and SOA formation.

**Table 4:** Major categories of biogenic volatile organic compounds (BVOCs) and their global average emission values (notes: Ref., references).

BVOCs Categories	Synthesis Method	Main Emission Plants Category	Contribution Rate	Global c (Tg C yr^−1^)	Ref.
1. Isoprenoids	MEP	Broadleaf trees	40–60%	400–600	[148,149,150]
2. Monoterpenes	MVA	Coniferous trees	25–40%	40–180	[151,152]
3. Sesquiterpenes	MEP and MVA	Tropical rainforest plants Herbaceous plants	5–15%	20–40	[153,154,155]
4. OBVOCs	LOX/MEP/MVA	Herbaceous plants; trees; grasses crops; flowers	10–25%	–280	[156,157]

**Table 5:** Mean concentrations of key biogenic volatile organic compounds (BVOCs) in different study areas and climatic conditions (Ref: references; -: no data).

Study Area	Seasonal Mean Concentrations (μg/m^3^)				Annual Mean Concentrations (μg/m^3^)	Ref.
	Spring	Summer	Autumn	Winter		
Isoprene						
Urban area of Beijing	1.83	2.82	1.48	0.43	1.66	[133]
Suburban areas of Tianjin	-	-	1.61	-	-	[158]
Urban area of Shenzhen	2.07	4.26	1.09-	1.06	-	[159]
Village of Guangzhou	6.69	-	4.26	0.40	3.41	[160]
Urban area of Shanghai	-	-	-	-	0.10	[161]
Urban area of Houston	-	-	-	-	1.92	[162]
Urban area of Lille	-	0.88	-	0.33	0.58	[162]
Zurich	-	0.49	-	0.24	-	[162]
α-Pinene						
Typical urban area of Beijing	0.05	0.11	0.18	0.04	0.10	[133]
Mountainous areas of Sichuan	-	-	-	-	0.30	[163]
Guangzhou Forest Park	-	1.12	-	-		[164]
β-Pinene						
Typical urban area of Beijing	0.05	0.003	0.006	0.002	0.02	[133]
Mountainous areas of Sichuan	-	-	-	-	0.30	[163]

### 4.3. Synthetic Pathways

Biogenic volatile organic compounds (BVOCs) in plants originate from three principal biosynthetic routes: the methylerythritol phosphate (MEP) pathway localized in plastids, the mevalonate (MVA) pathway operating in cytoplasmic compartments, and the lipoxygenase (LOX) pathway associated with peroxisomal membranes [165,166,167,168]. The MEP pathway predominates in higher plant terpenoid biosynthesis, generating monoterpenoids and diterpenoids via sequential condensation of isopentenyl diphosphate (IPP) and dimethylallyl diphosphate (DMAPP) precursors synthesized from glyceraldehyde-3-phosphate and pyruvate [169,170,171,172,173]. Counterintuitive to previous assumptions, the MVA pathway—traditionally associated with fungal sesquiterpenogenesis—exhibits functional conservation in plant cytoplasmic metabolism [174,175]. This pathway converts acetyl-CoA through enzymatic cascades to yield IPP/DMAPP, primarily fueling sesquiterpene and phytosterol biosynthesis, with emerging evidence confirming its collaborative role with the MEP pathway in generating specific monoterpenes under stress conditions [176,177]. The LOX pathway initiates the oxidative cleavage of polyunsaturated fatty acids (e.g., linolenic acid), producing jasmonate phytohormones and green leaf volatiles that orchestrate systemic defense responses against biotic stressors [178,179,180,181]. Therefore, studies on the components and formation pathways of BVOCs should be analyzed on a case-by-case basis, and the synergistic effects of multiple factors may result in emission scenarios that are quite different from a single analysis.

## 5. Influencing Factors of BVOC Emissions

**Figure 4:**
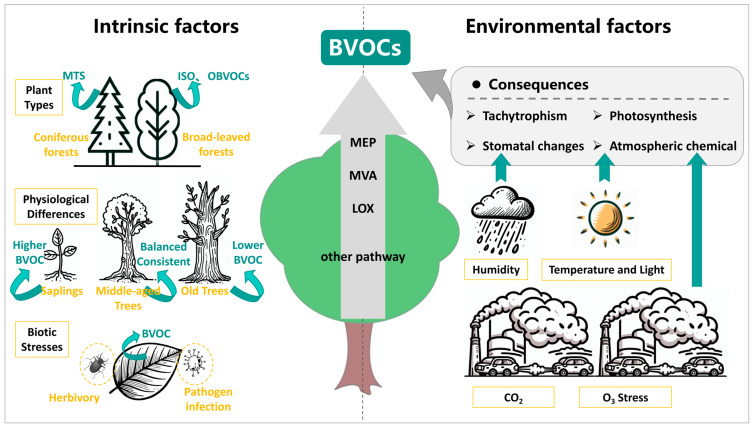
The effects of plant internal and environmental factors on biogenic volatile organic compounds (BVOCs)(notes: MTS, monoterpenes; ISO, isoprene; OBVOCs, oxygenated biogenic volatile organic compounds; MEP, Methylerythritol Phosphate Pathway; MVA, Mevalonate Pathway; LOX, Lipoxygenase Pathway; AVOCs, anthropogenic volatile organic compounds; CO_2_, carbon dioxide; O_3_, ozone).

The release of BVOCs is a complex, multifactor process influenced by both intrinsic plant factors (plant types, physiological differences, and biotic stresses) and environmental factors (temperature, light, humidity, CO_2_ concentration, and O_3_ stress), which may affect the release rate and release characteristics of BVOCs in plants [182]. These factors and their influence on BVOC emissions are shown in Figure 4.

### 5.1. Intrinsic Plant Factors Influencing BVOC Emissions

#### 5.1.1. Plant Types

Plant species are a key determinant of BVOC synthesis and release due to the differences in genetic control mechanisms and metabolic pathways, and the composition and release rate of BVOCs vary from different vegetation types [99]. Coniferous forests and broadleaf forests are two typical plant types, and their emission characteristics of BVOCs are different [183,184]. Coniferous forests mainly release monoterpenes and sesquiterpenes that may have more significant impacts on SOAs and the global climate system [185,186], while broadleaf forests mainly release short-chain BVOCs such as isoprene that are highly volatile and mainly affect photochemical smog and local O_3_ concentrations [187,188,189].

#### 5.1.2. Physiological Differences

The physiological state and development stage of plants also play a critical role in BVOC emissions. Younger trees generally emit BVOCs at higher rates, while mature trees emit higher absolute quantities of these compounds. Schade et al. [190] and Street et al. [191] found that young pine branches and leaves release BVOCs at a rate 2–3 times higher than mature branches and leaves. Kuzma and Fall [192] and Rasulov et al. [193] observed lower isoprene emissions from young leaves to mature leaves both in *Vicia faba* and *Populus alba*, respectively.

Stomatal conductance is another critical physiological trait that influences BVOCs’ synthesis and emission. As the primary pathway for BVOC emission, stomatal conductance affects the partitioning of BVOCs between the gas and liquid phases within leaves’ release [194,195,196,197]. Leaf structure also plays a role in modulating the effect of stomata on VOC emission [198,199].

#### 5.1.3. Biotic Stresses

Biotic stress, particularly pathogen infection and herbivore activity, can elevate BVOC emissions by 2–10-fold compared to unstressed plants, with emission profiles diverging based on stressor type (e.g., pathogen-induced oxygenated compounds vs. herbivore-induced terpenoids) [200,201]. Insect herbivory often increases BVOC emissions [202]; Joutsensaari et al. [203] noted that insect outbreaks could elevate BVOCs and potentially even BSOA emissions. However, severe pest or disease damage may impair physiological functions, such as reduced photosynthesis, resulting in a decrease in the overall production of BVOCs [204]. However, Litvak and Monson [205] found that while monoterpene synthesis in ponderosa pine increased following herbivore induction, the volatile losses from damage issues offset this increase, resulting in no significant net increase in the total monoterpene pool within the damaged needles.

### 5.2. Environmental Factors

#### 5.2.1. Temperature and Light

Temperature and light are critical environmental factors influencing the emission of BVOCs, as they influence plant enzymatic activity and physiological processes [206]. Generally, increases in temperature and photosynthetically active radiation (PAR) activate the enzymes responsible for BVOCs synthesis [207]. For instance, Li et al. [208] found that high temperatures could triple the monoterpenes emission flux. However, extreme temperatures may disrupt metabolic activities, leading to a decrease in emission rates. Loreto et al. [209] found that the isoprene emission from reed leaves increases with temperatures until 45 °C, after which emissions decline.

Different BVOC components exhibit varying dependencies on temperature and light. The synthesis of isoprene, which primarily occurs in chloroplasts, is directly dependent on the production of glyceraldehyde-3-phosphate (G3P) and energy from photosynthesis. Consequently, isoprene emissions are most influenced by light exposure [210,211,212]. Seasonal and latitudinal variations also affect emissions, with higher light intensities in summer and lower latitudes leading to stronger BVOC emissions.

#### 5.2.2. Humidity

Humidity indirectly influences BVOC emissions by influencing stomata conductance [213,214]. Higher humidity levels promote stomatal opening, facilitating BVOC emissions [215,216]. However, prolonged high humidity can reduce transpiration rates and induce stomatal closure, thereby reducing BVOC emissions [217]. BVOCs’ responses to humidity also vary by components. For instance, Ning et al. [218] found a positive correlation between α-pinene emissions and humidity, while isoprene emissions exhibited a negative correlation.

#### 5.2.3. Concentration of CO_2_

Changes in carbon dioxide (CO_2_) concentration influence photosynthesis and metabolic processes, subsequently affecting BVOC emissions. Elevated CO_2_ concentrations can inhibit BVOC emissions by suppressing photosynthesis, as observed in the reductions in monoterpenes and sesquiterpenes found by Constable et al. [219]. However, certain plants may exhibit increased BVOC synthesis under high CO_2_ concentrations, as found by Jasoni et al. [220] in onions. The duration of CO_2_ exposure is also critical. Rapparini et al. [221] found that short-term exposure can significantly suppress the emission of isoprenoids and monoterpenes, whereas long-term exposure had minimal effects.

#### 5.2.4. O_3_ Stress

The concentration of BVOCs plays a significant role in the formation of O_3_. Similarly, O_3_, as a potent oxidant, can influence the release of BVOCs through a series of complex physiological, biochemical, and genetic regulatory mechanisms [222,223,224,225]. Under increased O_3_ stress, plants may mitigate oxidative damage by increasing the release of BVOCs with strong antioxidant capacities. For example, isoprene emissions increase by 20–50% under acute O_3_ exposure (100 ppb for 4 h), as demonstrated in Populus tremula fumigation experiments [187]. Llusià et al. [226] also found that plants subjected to O_3_ fumigation exhibited increased net photosynthetic and total VOC emission rates. Li et al. [227] also discovered that exposure to O_3_ pollution increased BVOC emissions in the Yellow River Delta and the Yangtze River Delta by 13.9% and 4.9%, respectively. However, plant responses to O_3_ stress are also influenced by factors such as the exposure duration and the composition of BVOCs. Vo and Faiola [228] found that terpenoid emission rates decreased or remained unchanged after acute O_3_ exposure, and that sesquiterpenes exhibited a significantly higher response concentration to O_3_ than monoterpenes. Yuan et al. [75] found that under short-term high-concentration ozone stimulation, the emission of isoprene from leaves that were not severely damaged was promoted. However, prolonged exposure to high concentrations of O_3_ significantly inhibited isoprene emissions, resulting in a reduction of 40.4%. Additionally, the decrease in isoprene emissions from middle-layer leaves (61.1%) was significantly greater than that from upper-layer leaves (20.9%). Furthermore, this study also found that isoprene emissions were unrelated to stomatal conductance. The interaction between BVOC emissions and O_3_ is a dynamic process influenced by multiple factors. Understanding the impact of O_3_ on BVOC emissions requires consideration of multiple intrinsic and environmental variables.

## 6. Atmospheric Oxidation Mechanisms of BVOCs

**Figure 5:**
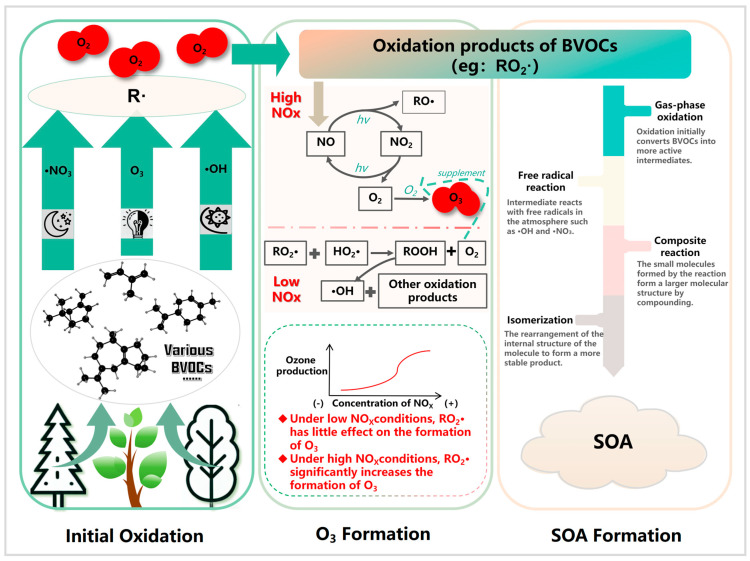
The initial oxidation reaction of biogenic volatile organic compounds (BVOCs) and the pathway diagram of their formation of ozone (O_3_) and secondary organic aerosols (SOAs) (notes: for the corresponding abbreviations in the chart, please refer to nomenclature (Table S1)).

The release of BVOCs has a significant impact on atmospheric chemistry. Once released into the atmosphere, these BVOCs undergo a complex series of photochemical reactions that produce and affect the corresponding free radicals, which influence the formation of O_3_ and SOA in the atmosphere. Understanding the role of BVOCs in atmospheric chemistry and their transformation processes (Figure 5) is critical for comprehending atmospheric chemistry, air quality, and climate change implications [229,230].

### 6.1. Initial Oxidation Reactions

The initial oxidation reaction pathways of BVOCs in the atmosphere include reactions with hydroxyl radicals (•OH), •NO_3,_ and O_3_ [231,232,233,234].

The primary source of •OH in the troposphere is from the decomposition of O_3_ under ultraviolet radiation, which generates excited oxygen atoms O(^1^D). This process results in high concentrations of •OH during the daytime [235,236,237]. Consequently, during the day when ultraviolet intensity is high, highly reactive BVOCs frequently undergo addition and oxidation reactions with •OH. The hydrogen atoms in carbon-based BVOCs are abstracted by •OH to produce alkyl radicals (R•) and H_2_O [238,239]. At night, in the absence of light, the reactions between •OH and BVOCs are greatly inhibited, while •NO_3_, having high stability, becomes the primary radical reacting with BVOCs during nighttime [240,241,242]. Mogensen [243], Xu [244], and Waring and Wells [245] corroborate this mechanism. Since the reaction between O_3_ and BVOCs is not dependent on light and is highly reactive, at night, •NO_3_ radicals (derived from O_3_ + NO_2_) are typically the primary oxidants for BVOCs in NO_x_-rich environments. However, in regions with extremely low NO_x_ levels (e.g., pristine forests), the scarcity of •NO_3_ shifts the dominant oxidation pathway to reactions between O_3_ and unsaturated BVOCs (e.g., isoprene and monoterpenes), generating carbonyl compounds and sustaining nighttime radical chemistry [246,247,248]. Atkinson [249], Docherty [250], and Kroll [251] found that the oxidation of organic compounds by O_3_ differs from radical oxidation because BVOCs can be cleaved by O_3_ to form carbonyls and energetically excited carbonyl oxides. These intermediates then decompose via the hydroperoxide channel to produce •OH and R•. The organic peroxyl radicals (RO_2_•) from the primary oxidation of BVOCs are part of the radical cycling that controls atmospheric oxidative capacity, leading to the subsequent formation of O_3_ and SOA [252].

### 6.2. O_3_ Formation

Numerous studies have demonstrated that BVOCs play a critical role in the formation of O_3_ through photochemical reactions, and this role largely depends on the concentration of nitrogen oxides (NO_x_) [231,253,254,255]. According to the studies by Xie [256] and Kim et al. [257], biogenic and anthropogenic VOCs, especially the photochemical reactions of isoprene, significantly increase the formation of local O_3_.

Under high-NO_x_ environments, RO_2_• reacts mainly with NO to produce new oxidation products (RO•, NO_2_). The newly formed NO_2_ undergoes photolysis (NO_2_+ hν → NO + O(^3^P)), followed by O(^3^P) + O_2_ → O_3_, while RO• decomposes to yield carbonyl compounds (e.g., HCHO) that further sustain radical cycling [258,259]. Simon et al. [260] found that to a certain extent, the more NO_x_ there is, the higher the rate of O_3_ production, which is largely dependent on the NO_2_/NO ratio and the amount of BVOCs available. Zhang et al. [261] found that when in high-NO_x_ conditions, the increase in BVOCs will effectively promote radical cycling and enhance the level of radicals, which to some extent may lead to an increase in O_3_.

In contrast, the reaction of RO_2_• with hydroperoxy radicals (HO_2_•) is more important in low NO_x_ concentration environments, where NO_x_ is not a major limiting factor. RO_2_• reacts with HO_2_• to produce peroxides (e.g., organic hydroperoxides (ROOH)), and these reactions do not usually result in the direct production of NO_2_, and therefore contribute less to O_3_ production. In this environment, the rate of O_3_ production is limited by the concentration of VOCs and the rate of reaction [262,263]. Orlando and Tyndall [252] and Iyer et al. [264] found that under low NO_x_ conditions, RO_2_• reacts mainly with HO_2_•, a reaction that is very important in the atmosphere and is the end of O_3_ production, manifesting itself in the fact that BVOCs produce less O_3_ or even consume O_3_.

### 6.3. SOA Formation

BVOCs are crucial precursors for the formation of SOA [265,266,267]. SOA consists of fine particulate matter formed through a series of complex chemical transformations of BVOCs, significantly impacting atmospheric climate and human health [268,269]. Among these, isoprene, terpenes, and sesquiterpenes are the most common BVOC precursors [270,271].

The formation process of SOA involves multiple reaction steps. BVOCs first undergo gas-phase oxidation, generating RO_2_•. These radicals can react through different pathways to produce secondary products, such as reacting with NO to form NO_2_ and R•, reacting with HO_2_• to produce ROOH, or reacting with other RO_2_• to form peroxides (ROOR) and other organics [272,273]. These organics include low-volatility organic compounds (LVOCs) or extremely low-volatility organic compounds (ELVOCs) [274,275]. Concurrently, these LVOCs and ELVOCs can further grow into larger sizes through processes like complexation, isomerization, and condensation nucleation, ultimately forming SOAs [276,277]. This process is also influenced by environmental factors such as temperature, humidity, and NO_x_ concentrations [278,279]. BVOCs are transformed into SOA through complex chemical processes in the atmosphere, influenced by multiple environmental factors. BVOCs such as isoprene, terpenes, and sesquiterpenes undergo oxidation, radical reactions, condensation and adsorption complex reactions, and isomerization, forming SOAs through multiple steps. The formation and evolution of SOA not only affect the balance of atmospheric chemistry but also have significant implications for air quality and human health.

### 6.4. Synergistic Effects of BVOCs with AVOCs

Both AVOCs (such as NO_x_ and toluene) and BVOCs (such as isoprene and α-pinene) participate in atmospheric photochemical reactions that exacerbate O_3_ production [280] and promote the condensation of ELVOCs into SOA [281]. In high-NO_x_ environments, the reactivity of BVOCs is enhanced; for instance, BVOCs accounted for 56% of emissions during summer nights in Taipei, thereby extending the photochemical chain when combined with AVOCs. In semi-urban areas of Turkey, the cumulative impact of traffic emissions (AVOCs at 16%) and vegetation releases (BVOCs at 38.6%) has resulted in peak summer O_3_ levels, with both contributing synergistically to over 60% of ozone formation potential [282,283]. Li et al. [284] found, through analyzing results from different simulation scenarios, that AVOCs promote O_3_ production by interacting with BVOCs, with the effect being most pronounced from noon to evening. Choi et al. [285] found, through combining Proton Transfer Reaction Time of Flight Mass Spectrometer (PTR-ToF-MS) and the Aerodyne High Resolution Time of Flight Aerosol Mass Spectrometer (HR-ToF-AMS) data, that BVOCs (e.g., monoterpenes) and AVOCs (e.g., toluene) cooperatively drive SOA production through daytime OH oxidation and nighttime NO_3_ chemistry, with aqueous-phase reactions dominating under high humidity and gas-phase partitioning prevailing in polluted air masses. Ghirardo et al. [286] found that BVOCs (e.g., sesquiterpenes) synergistically enhance SOA formation with AVOCs through NO_x_-mediated oxidation pathways, where anthropogenic NO_x_ promotes the conversion of both BVOCs and AVOCs into low-volatility products and aerosol aging processes involving multi-phase reactions (e.g., acid-catalyzed polymerization), with their combined contributions amplified under urban pollution and plant stress conditions.

In high-NO_x_ environments, the O_3_ and SOA formation efficiency of BVOCs is significantly enhanced. Moreover, emission control strategies targeting anthropogenic sources, such as reducing VOCs or NO_x_, can either amplify or diminish the contribution of biogenic sources. Notably, under carbon neutrality pathways, biogenic contributions may surpass those from anthropogenic sources, highlighting the dual challenges of regional collaborative emission reduction and climate change.

## 7. Atmospheric Impacts of BVOCs

As critical mediators of biosphere–atmosphere interactions, biogenic volatile organic compounds (BVOCs) profoundly influence air quality, climate systems, and public health through complex atmospheric oxidation pathways [287,288].

### 7.1. Ozone Formation Potential (OFP) and Climate Feedbacks

The OFP quantifies the mass-specific contribution of BVOCs to ozone production. The O_3_ formation potentials of isoprene and monoterpenes were 0.05–57.21 and 0.003–72.35 ppb [289]. In addition to this, aromatic hydrocarbons such as toluene and o-xylene and olefins such as ethylene and propylene also contribute significantly to OFP [290]. This potential is amplified in the urban–rural boundary layer, where BVOC-NO_x_ synergies elevate surface O_3_ by 13–16 ppb [291]. For instance, emissions of reactive olefins (e.g., isoprene) in Zhengzhou during the summer combine with traffic NO_x_ to result in O_3_ concentrations that exceed China’s Grade II standard (93.3 ppbv) by more than 50% [292].

### 7.2. Secondary Organic Aerosol Formation Potential (SOAP) and Environmental Impacts

The SOAP characterizes the nucleation efficiency of BVOC oxidation products. The SOA values for isoprene and monoterpenes are 0.0002–0.21 and 0.0006–10.46 μg/m^3^, respectively [289]. Sesquiterpene oxidation products play an important role in the formation and oxidative chemistry of atmospheric SOA, especially during the spring recovery period [229]. BSOAs exhibit different diurnal formation mechanisms. NO_x_-induced SOA production dominates at night, and its impact on the environment is influenced by solar radiation, temperature, and humidity [293]. It has been shown that nocturnal terpene ozonolysis with high NO_x_ levels can significantly contribute to secondary organic aerosol formation and organic nitrate formation through anthropogenic–biotic interactions [244].

From 2000 to 2019, the contribution of secondary species to 2.5-micrometer particulate matter (PM2.5) in the Pearl River Delta, China, stabilized at about 80%, with SOAs and secondary inorganic aerosols (SIAs) contributing equally to fine particulate pollution [294]. The presence of SOAs in the atmosphere has implications for climate and health. Respiratory diseases, reduced visibility, haze, deterioration of air quality leading to environmental pollution, etc., are the result of the presence of organic species in atmospheric aerosols [295].

## 8. Future Research Challenges

While significant progress has been made in understanding BVOC emission mechanisms, characterization methods, and their atmospheric impacts, several challenges remain:

(1) Modeling methods such as Guenther, MEGAN, and BEIS are research methods that overcome spatial and temporal limitations. However, accurate and credible emission prediction models of BVOCs require a substantial amount of high-precision and readily accessible measurement data.

(2) Free radical reactions play a key role in the atmospheric transformation process of BVOCs, directly influencing the levels of O_3_ and SOA. Nevertheless, the specific reaction pathways in the oxidation stage of BVOCs, as well as the details and mechanisms of the dynamic changes in free radicals, remain poorly understood.

(3) The emission of BVOCs is affected by a multitude of factors, many of which do not individually influence the emission of BVOCs. There is a dearth of systematic studies on the multiple synergistic effects of the internal and external factors on the emission mechanisms of BVOCs and their long-term impacts.

Given these challenges, the following three research areas are particularly important for future BVOC studies: (1) the development of high-precision, low-cost, and easy-to-operate methods for rapid measurements of BVOCs; (2) an in-depth study of the atmospheric chemical reaction mechanism between BVOCs and free radicals; and (3) an investigation into the synergistic effects of multiple factors on the emission mechanism of BVOCs.

## 9. Recommendations and Prospects

### 9.1. Recommendations of BVOC Prevention and Control

(1) Formulate BVOC management strategies or guidelines:

Among the world’s major economies, the EU’s Clean Air Quality Directive, the U.S. Clean Air Act, and China’s Comprehensive Control Program for Volatile Organic Compounds in Key Industries all have stringent controls on the emission of anthropogenic VOCs. However, all of these programs merge and unify the management of anthropogenic sources of VOCs, such as vehicles, industry, and construction materials, and do not provide effective guidance on BVOCs. Meanwhile, the guidance of each country is basically formulated based on its own situation and lacks universality and popularization. It is recommended that developed countries with high forest cover, such as the U.S. and Canada, and developing countries with high forest resources, such as China and Brazil, cooperate to incorporate the management of BVOCs into an integrated air quality management framework, focusing on their synergistic effects with anthropogenic pollutants, and to further improve the global VOCs pollution situation.

(2) Establish BVOC emission control indicators based on multi-dimensional parameters:

In contrast to the relatively straightforward and predictable emissions observed in laboratory settings, the release of BVOCs in natural environments is a complex and variable process. It is therefore evident that a single indicator cannot be relied upon for the quantitative control of BVOC emissions. By cross-analyzing multi-dimensional parameters through big data, accurately identifying the emission characteristics of different BVOC sources, and highlighting the major items, a more scientific and targeted BVOC emission control indicator can be formulated. This approach helps to avoid the inaccuracy and significant errors of single indicators, optimize the use of resources, and reduce unnecessary economic losses and control costs.

(3) Dynamically adjust BVOC management based on temporal and spatial characteristics of BVOC emissions:

BVOC emissions exhibit significant seasonality and diurnal variations over time and are influenced by geographical and climatic conditions spatially. Additionally, the synergistic pollution effects with other pollutants like anthropogenic NO_x_ vary. Therefore, it is essential to implement differentiated control strategies based on the temporal and spatial characteristics of specific regions and adjust BVOC management strategies dynamically according to local conditions. This makes policies more flexible and scientific, enhancing the effectiveness and specificity of regional management.

### 9.2. Prospects of BVOC Monitor Strategies

(1) Develop more efficient, convenient, and low-cost BVOC measurement technologies:

As previously stated, a rapid sampling method for BVOCs that is highly accurate, straightforward to operate, and cost-effective is the foundation for obtaining high-quality detection data and conducting related research. The Guangzhou Institute of Geochemistry, Chinese Academy of Sciences (GIGC) devised a semi-open dynamic chamber system for measuring BVOCs. This system can reduce the time required to reach a steady state at higher flow rates, thereby minimizing adsorption loss. Nevertheless, the adsorption loss for macromolecular compounds remains above 30%, indicating a need for further optimization [296]. Electrochemical sensors are employed for the measurement of atmospheric NO_2_ due to their low cost, portability, and flexibility, which can also be applied to the measurement of BVOCs [297]. Laser-induced fluorescence tracking has also been employed for the sampling and measurement of BVOCs, although the technology is not yet fully developed [298]. It is therefore feasible to pursue the optimization of existing methods or the adoption of techniques from other fields as avenues for the development of more efficient, convenient, and cost-effective measurement techniques for BVOCs.

(2) Develop BVOC monitoring and prediction technologies based on high temporal and spatial resolution big data models:

The advent of new information science and technology has opened up new avenues for research in the field of environmental management. By extending the aforementioned laser-induced fluorescence tracking technology, the regional dissemination of environmentally friendly fluorescent probes, and the laser-induced generation of remotely sensed data through the use of UAVs, in conjunction with ground-based observation data, a comprehensive database of high temporal and spatial resolution BVOC emissions can be formed. The application of machine learning and deep learning algorithms can enhance the synergistic effects of multiple factors, optimize existing BVOC emission models, and facilitate the development of new prediction models. A map of BVOC emission characteristics in different regions can be constructed, allowing region-specific control strategies to be formulated and precise management to be achieved.

(3) Promote the selection of low-BVOC-emitting tree species in urban greening and ecological restoration:

In urban greening and ecological restoration, besides considering the ornamental value and ecological function of trees, selecting low-BVOC-emitting tree species can help optimize air quality while maintaining ecological function and aesthetic values.

(4) Artificially control precursor substance emissions:

The strengthening of emission controls of precursors such as O_3_ and SOA can indirectly reduce the emission level of BVOCs, reducing secondary chemical reactions that lead to O_3_ and SOA formation.

## Figures and Tables

**Table 1:** Comprehensive comparisons of biogenic volatile organic compounds (BVOCs) emission measurement methods.

Measurement Methods	On-Line	Off-Line	
Main technologies	Direct analysis: PTR-MS [32] FTIR [33] GC-FID [34] Portable GC/MS [35] E-noses [36]	Sampling: sampling bags, adsorption tubes, sampling tanks	Analysis: GC-MS [37] TDS-GC/MS [38] TCT-GC/MS [39] TD-GC/TOF-MS [40] SPME-GC/MS [41]
Advantages	Real-time monitoring; high temporal resolution; high sensitivity; high degree of automation	Lower equipment costs; high flexibility; diverse and detailed analysis	
Disadvantages	Expensive equipment; complexity; high on-site requirements	Time delay; lower temporal resolution; human error	
Comprehensive comparison	Precision and sensitivity: on-line > off-line Operational complexity: on-line > off-line		

**Table 2:** Biogenic volatile organic compounds (BVOCs) analytical techniques comparison (notes: Ref., references).

Analysis Technique	Detector Type	Sensitivity	Time Resolution	Advantages and Disadvantages	Applicable BVOC Types	Ref.
PTR-MS	Mass spectrometry	ppt- ppm	Real-time analysis	Sensitivity and cost	Most BVOCs	[42,43]
FTIR	Pyroelectric detector or photoconductive detector	ppb- ppm	Minutes to hours	Versatility and sensitivity	Polar BVOCs	[44,45]
GC-FID	Flame ionization detection	ppt	Minutes	High speed and limitations	Nonpolar BVOCs	[34,46]
E-noses	Gas sensors	ppb- ppm	Minutes	Speed and resolution	Trace BVOCs	[36,47]
Portable GC/MS	Mass spectrometer	ppb	Minutes	Portability and limitations	Most BVOCs	[48,49]
GC-MS	Mass spectrometer	ppt	Minutes to hours	Precision and complexity	Most BVOCs	[37,50]
TD-GC/MS	Mass spectrometer	ppb	Minutes	Sensitivity and time-consuming	Trace BVOCs	[38,51]
TCT-GC/MS	Mass spectrometer	ppb	Minutes	Precision and requirements	Complex BVOCs	[39,52]
TD-GC/TOF-MS	Time-of-flight mass spectrometer	ppb	Minutes	Resolution and cost	Trace BVOCs	[40,53]
SPME- GC/MS	Mass spectrometer	ppb	Minutes	Simplicity and sensitivity	Most BVOCs	[41,54]

## Data Availability

No new data were created or analyzed in this study. Data sharing is not applicable to this article.

## Supplementary Materials

The following Supporting Information can be downloaded at: https://www.mdpi.com/article/10.3390/toxics13050364/s1, Table S1: Nomenclature.
