# Supplementary material for: A Review of Biogenic Volatile Organic Compounds from Plants: Research Progress and Future Prospects
Source: Toxics. 2025 Apr 30;13(5):364. doi: 10.3390/toxics13050364 (PMC12115729; doi:10.3390/toxics13050364)
Supplement: Supplementary file 1 [file toxics-13-00364-s001.zip › toxics-3485867-supplementary.pdf]

Keywords

Biogenic volatile organic compounds (BVOCs); Air pollution; Ozone(O3); Secondary organic aer-osol (SOA); Sampling analysis; Reaction mechanisms

Nomenclature

BVOCs	Biogenic volatile organic compounds	O_3	Ozone
SOA	Secondary organic aerosol	VOCs	volatile organic compounds
AVOCs	anthropogenic volatile organic compounds	BSOA	biogenic secondary organic aerosols
GC-MS	gas chromatography-mass spectrometry	TDS-GC-MS	thermal desorption-gas chromatography-mass spectrometry
$\bullet NO_3$	nitrate radicals	SBVOCs	soil BVOCs
OBVOCs	Oxygenated Biogenic Volatile Organic Compounds	MEP	methylerythritol phosphate pathway
U-BVOCs	the BVOCs emitted from plants in urban areas	N-BVOCs	the BVOCs emitted from plants in non-urban areas
MVA	mevalonate pathway	LOX	lipoxygenase pathway
IPP	isopentenyl diphosphate	DMAPP	dimethylallyl diphosphate
PAR	photosynthetically active radiation	G3P	glyceraldehyde-3-phosphate

CO_2	carbon dioxide	•OH	hydroxyl radicals
^1D	excited oxygen atoms O	R•	alkyl radicals
RO_2•	organic peroxyl radicals	NO_x	nitrogen oxides
NO_2	nitrogen dioxide	HO_2•	hydroperoxy radicals
ROOH	organic hydroperoxides	ROOR	peroxides
LVOCs	low-volatility organic compounds	ELVOCs	extremely low-volatility organic compounds
PTR-MS	proton transfer reaction-mass spectrometry	FTIR	fourier transform infrared spectroscopy
GC-FID	portable gas chromatography-flame ionization detector	SPME-GC/MS	solid-phase microextraction sampling combined with gas chromatography-mass spectrometry
E-noses	electronic noses	TCT-GC-MS	automatic thermal desorption-gas chromatography-mass spectrometry
TD-GC/TOF-MS	thermal desorption gas chromatography-time-of-flight mass spectrometry	PM2.5	2.5-micrometer Particulate Matter
Guenther	Guenther model	MEGAN	model of emissions of gases and aerosols from nature

BEIS	biological source emission inventory system model	NO	nitric oxide
NCAR	the National Center for Atmospheric Research	EPA	the United States Environmental Protection Agency
EMBE	the empirical model of BVOC emissions	PSEM	Plant Specific Emission Model
OFP	Ozone Formation Potential	SOAP	Secondary Organic Aerosol Formation Potential
SIA	Secondary inorganic aerosol	GIGC	The Guangzhou Institute of Geochemistry, Chinese Academy of Sciences
HAPs	Hazardous air pollutants	BAT	Best Available Technology
